# A Self-Adjusting Search Domain Method-Based Genetic Algorithm for Solving Flexible Job Shop Scheduling Problem

**DOI:** 10.1155/2022/4212556

**Published:** 2022-10-10

**Authors:** Bin Li, Xuewen Xia

**Affiliations:** ^1^College of Computer, Minnan Normal University, Zhangzhou 363000, China; ^2^Key Laboratory of Data Science and Intelligence Application, Minnan Normal University, Zhangzhou 363000, China; ^3^Key Lab of Intelligent Optimization and Information Processing, Minnan Normal University, Zhangzhou 363000, China; ^4^College of Physics and Information Engineering, Minnan Normal University, Zhangzhou 363000, China

## Abstract

As a nondeterministic polynomial (NP) problem, the flexible job shop scheduling problem (FJSP) is a difficult problem to be solved in terms of finding an acceptable solution. In last decades, genetic algorithm (GA) displays very promising performance in the field. In this article, a hybrid algorithm combining global and local search with reinitialization (GLRe)-based GA is proposed to minimize makespan for FJSP. The solution of FJSP is conveniently represented by a double-layer chromosome representation method, which is convenient for subsequent genetic operations, that is, sorting of operations and selection of machines. Two strategies of choosing the job with the most remaining operations (CRO) and 6-dimensional variable determined search position (6D-VSP) are proposed as two components for GA, which are applied to generate a population with superior quality and reduce the global search space during the initialization stage. At the same time, in order to prevent the loss of diversity during evolution, a reinitialization strategy is introduced in the later stage of evolution to adaptively adjust the search domain of the problem. Finally, two sets of benchmark data are tested. The experimental results demonstrate the accuracy and effectiveness of the GLRe proposed in this article for solving FJSP.

## 1. Introduction

Manufacturing is an industry that converts certain resources into products that people can use through the manufacturing process. It basically covers all industries. The development of manufacturing is an important factor in measuring the level of national development. As a key part of the manufacturing industry, job shop scheduling (JSP) has a positive impact on the competitiveness of enterprises. At the same time, the study of JSP has certain reference significance for the research of scheduling problems in other fields.

As one of the sub-processes of production planning, scheduling plays an important role in modern manufacturing systems. In this field, the JSP is recognized as one of the most basic, typical, and toughest problems to be solved. As a nondeterministic polynomial (NP) and combinatorial optimization problem (COP) [1], JSP has gradually gained more attention in recent decades.

The description of the classic JSP is as follows. A range of machines needs to process a group of jobs with unequal operations. Each operation takes a certain amount of time to process on a machine [2]. In order to optimize for a certain objective, a correct sequence of operations must be determined, and a suitable machine for each operation must be arranged. However, for increasing the productivity in the actual production process, one machine is capable of processing different jobs, and each type of operation is available to be performed on one or more different machines. This type of problem is known as flexible job shop scheduling problem (FJSP) [3], which can be regarded as a branch of the classic JSP [4].

In FJSP, restrictions on processing machines are relaxed, which is more consistent with conditions of an actual production. Each operation can be executed on available machines, which means that each machine has the ability of processing one or more different operations. The same operation takes different processing times on different machines. Therefore, it is more difficult to be solved than classic JSP. FJSP is also NP-hard, just like classic JSP [5].

On the one hand, the complexity of FJSP is manifested in the need to sequence jobs and make decisions about the machines that process an operation. On the other hand, the description of the complexity of FJSP is the evaluation of the search space, which is also a test of the algorithm's fast search ability.

For a simple JSP with the characteristic of randomly generating schedules, the total number of operations (*p*) and the number of machines (*m*) are fixed, and *m*^*p*^ sequences can be generated. Each sequence is a possible solution. Therefore, under the premise of flexible scheduling, the problem will become more complicated. The algorithm cannot handle a huge amount of data in a short time, and it is difficult to solve it quickly. The time complexity and space complexity are proportional to the problem size. At the same time, it is inversely proportional to the efficiency of algorithm execution. It has been demonstrated in a published article that the size of the search space increases exponentially with the scale of the problem.

In order to solve FJSP, scholars have proposed various algorithms from different perspectives. However, there are some common shortcomings of these algorithms, such as large search space and the inability to solve quickly.

The goal of FJSP is to obtain a reasonable scheduling sequence by allocating resources. This characteristic is well matched with the concept of chromosomes in genetic algorithm. A sequence is obtained after genetic operations of a chromosome. An optimal scheduling scheme is finally obtained by decoding. Therefore, this article chooses genetic algorithm as the basic algorithm.

Meanwhile, in an algorithm, a reasonable initialization method can reduce the search space to a certain extent. It should also reduce the time complexity of the algorithm and shorten the calculation time. A good algorithm should also avoid the population falling into local optima at a later stage in evolution to the greatest extent possible. Therefore, it is necessary to design a more efficient and accurate algorithm for FJSP.

In this article, we propose an improved hybrid algorithm combing global and local search with reinitialization (GLRe)-based genetic algorithm (GA) to solve FJSP. Firstly, considering there are two issues that need to be dealt with, that is, operations sequence (OS) and machine selection (MS), we adopt a double-layer gene chain structure in encoding and decoding phrases. Moreover, taking into account the load capability of each machine, a new initialization approach is proposed to reduce the search space of the problem in the initialization stage. After a population falls into a local optimum, some chromosomes in the population are reinitialized aiming to raise the diversity of the population. Meanwhile, different crossover and mutation operation strategies are used.

This article is organized as follows.  Section 2 gives a detailed description of FJSP. Some relevant literature addressing FJSP is listed in Section 3.  Section 4 describes the processing of population initialization, chromosome encoding and decoding schemes, selection, and other genetic operations. In Section 5, the performance of this algorithm is introduced and analyzed through comparative experiments. In Section 6, some final conclusions are provided.

## 2. Problem Formulation

FJSP can be classified into partial FJSP (P-FJSP) and total FJSP (T-FJSP). This article conducts research on P-FJSP. The FJSP is formulated as a set of *n* jobs *J* = {*J*_1_, *J*_2_, *J*_3_, ..., *J*_n_} that need to be processed on *m* machines *M* = {*M*_1_, *M*_2_, *M*_3_, ..., *M*_m_}. Each job *J*_i_ consists *u* operations *O* = {*O*_i1_, *O*_i2_, *O*_i3_, ..., O_iu_}. *O*_*i,j*_ denotes the *j-th* operation of *J*_*i*_. Each operation needs to select one machine from the feasible machine group to process. In addition, the processing sequence of all operations needs to be determined to minimize the makespan, which is the total operation time from the start of processing to the completion of all operations. There are some restrictions on the assignment between jobs and machines. Below, the optimization objective function and constraints are given, and related concepts are explained.

Obj.

(1) $$\begin{array}{c} f=min\;\left(\overset{n}{\underset{i=1}{\sum }}\overset{u}{\underset{j=1}{\sum }}S_{i,j,k}+t_{i,j,k}\right)\text{,} \end{array}$$

s.t.

(2) $$\begin{array}{c} t_{i,j,k}>0\text{,} \end{array}$$

(3) $$\begin{array}{c} \overset{m}{\underset{k=1}{\sum }}X_{i,j,k}\geq 1\text{,} \end{array}$$

(4) $$\begin{array}{c} S_{i,j,k}+t_{i,j,k}⩽S_{i,j+1,k}\text{,} \end{array}$$

(5) $$\begin{array}{c} \overset{n}{\underset{i=1}{\sum }}\overset{u}{\underset{k=1}{\sum }}X_{i,j,k}=1\text{,} \end{array}$$

(6) $$\begin{array}{c} T_{i,i,k}-P_{i,j,k}=t_{i,j,k}\text{,} \end{array}$$

(7) $$\begin{array}{c} x_{i,j,k}=\left\{\begin{array}{cc} 1\text{,} & if\;machine\;k\;can\;process\;O_{i,j}\text{,}\\ 0\text{,} & otherwise\text{,} \end{array}\right. \end{array}$$

where the objective of scheduling is to minimize the makespan of all operations. The start time of an operation *O*_*i,j*_ processing on machine *k* is denoted by *S*_*i,j,k*_. *t*_*i,j,k*_ means the processing time of the operation *O*_*i,j*_ on the machine *k*. *T*_*i,j,k*_ is the time point when the current operation *O*_*i,j*_ completes processing. *P*_*i,j,k*_ denotes the time point when operation *O*_*i,j*_ begins processing.

Equation (2) indicates that the processing time of each operation is greater than 0. Equation (3) represents that each operation can be run on at least one machine. Equation (4) shows that all operations are performed in a predefined order. Equation (5) means that when a machine is processing a certain operation, other operations cannot be performed at the same time. Equation (6) explains that each operation cannot be interrupted until the processing is completed and the transportation time between operation and machine is negligible.

## 3. Literature Review

Brucker and Schile [6] firstly took the machining flexibility into account in 1990. However, their proposed polynomial graph algorithm is not efficient for larger instances and complex FJSP. With the development of methods for solving NP-hard problems, it is found that heuristic and meta-heuristic algorithms display more favorable performance than these traditional methods [7–9]. Thus, in recent decades, it has been widely used for solving FJSP.

Nouri et al. [10] used a neighborhood-based genetic algorithm (NGA) to solve FJSP in which scheduler agents and cluster agents are used to guide the search process. With the goal of minimizing the maximum completion time of all jobs, Jiang and Zhang [11] proposed a variable neighborhood search method based on gray wolf optimization, which further strengthen the exploration of the algorithm itself. Xing et al. [12] put forward a knowledge-based ant colony optimization (KBACO) algorithm for FJSP. The model of KBACO learns from ant colony optimization and applies the acquired knowledge to guide the current search process.

Zhang et al. [13] proposed an efficient hybrid method that combines particle swarm optimization (PSO) algorithm and tabu search (TS) algorithm to improve the efficiency of local and global searches and find a near-optimal scheduling scheme. Gao et al. [14] used a variable neighborhood descent (VND) with GA-based algorithm to find assignable time intervals for the deleted operations based on the concept of earliest and latest event time. Li and Gao [15] presented a hybrid algorithm for FJSP based on GA and TS, which uses the characteristics of the two algorithms to control the global and local search processes, respectively, and balance intensification and diversification well. Zhao et al. [16] proposed a self-learning discrete Jaya algorithm (SD-Jaya) to solve the energy-saving distributed heterogeneous flow shop scheduling problem (FSP). Taking into account energy consumption, Zhao et al. [17] proposed a two-stage cooperative evolutionary energy-saving scheduling algorithm (TS-CEA) to solve FSP.

In terms of initialization mode and genetic operations, scholars proposed different strategies. Kacem et al. [18] presented a new assignment localization (AL) model that assigns tasks based on the load capacity of each machine. Zhang et al. [19] proposed a global and local selection approach (GL) based on GA, which takes into consideration the total load capacity of all machines. Pezzella et al. [20] put forward an integration strategy to generate the initial population and select individuals to breed new individuals. Mahmudy [21] proposed an improved real-coded genetic algorithm (IRCGA) that uses real vectors as chromosome representations better helps the algorithm in searching. Amjad et al. [22] proposed a new formula based on a feedback mechanism. According to the formula, the probability of crossover and mutation can be adjusted automatically in the process of evolution. Chen et al. [23] investigated a self-learning genetic algorithm (SLGA) based on reinforcement learning, which can adjust key parameters intelligently. Zhou et al. [24] used an adaptive differential evolution algorithm with the goal of minimizing the manufacturing period. The control parameter values and mutation operators in the algorithm are adaptively selected according to their historical performance. Based on the reinforcement learning mechanism, Zhao et al. [25] proposed a cooperative water wave optimization algorithm (CWWO) in the framework of VNS. The algorithm combines path relinking and VNS method as an improved interrupt operator to enhance local search ability.

As discussed above, multiple categories of meta-heuristic algorithms have been used to solve FJSP successfully [26]. Due to the better performance and greater generality of GA, it has gained great attention [27]. Furthermore, extensive studies have demonstrated that GA has superior performance for the quality of the solution. However, in the existing literature, there is a common shortcoming that the makespan is too large after initialization, which leads to an excessively large space for searching and seriously affects the quality of the final solution. Moreover, at the later stage of evolution, the diversity of chromosome in the population declines seriously. As a result, the population nearly stops evolving.

## 4. The Proposed Algorithm

As a widely used heuristic algorithm, GA has been successfully applied in solving COPs [28]. Unlike traditional deterministic methods, GA uses a population composed of a certain number of individuals to perform an optimizing process. Note that, each individual in GA is called a chromosome denoting a candidate solution. During the search process, new chromosomes are generated through crossover, mutation, and selection operations, and genes on the chromosomes are changed to generate new chromosomes. Newly generated individuals are continuously added to the mating pool for evolution. When a given number of generations is reached, the final solution can be obtained by decoding the optimal individual.

### 4.1. Chromosome Representation

When using GA to optimize a problem, the first issue that needs to be dealt with is designing a proper chromosome representation method. Considering distinct properties of FJSP, this article proposes a double-layer chromosome representation. The details of the representation are described as follows based on a simple example. A simple instance with three jobs and four machines of P-FJSP is shown in Table 1, where columns 1 and 2 correspond to jobs and operations, respectively, and columns 3–6 correspond to machines. The numbers in the table represent the required time that a machine processes an operation. The symbol “-” in the table indicates that a machine cannot process a corresponding operation. For example, for Job_1_, it contains two operations, the first operation can be performed on the 2nd, 3rd, and 4th machines, and the second operation can be performed on the 1st, 3rd, and 4th machines. The essence of FJSP is the two sub-problems of OS and MS [29]. Therefore, the double-layer chromosome representation improves the traditional lengthy single-layer method, with the first layer representing OS and the second layer representing MS. This representation makes encoding and decoding easier and avoids the formation of illegal chromosomes and unnecessary repair mechanisms.

### 4.2. Operation Sequence Part

**Figure 1:**
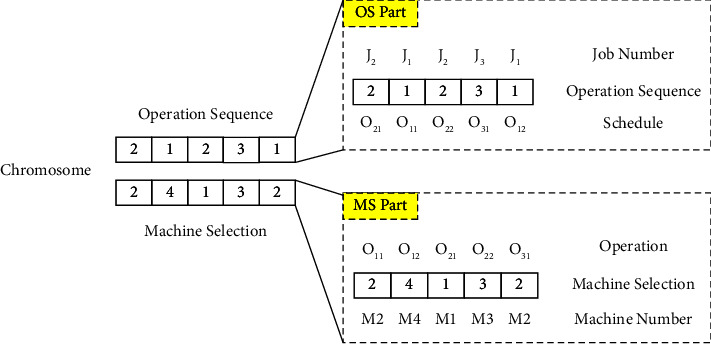
A chromosome generated from Table 1.

A feasible chromosome generated according to Table 1 is shown in Figure 1. Due to the double-layer gene chain structure adopted, the length of a chromosome is equal to the number of all operations. In the OS part, the first “2” represents the first operation of the second job, which can be represented by *O*_21_. The second number “1” expresses the first operation of the first job, represented by *O*_11_. The second occurrence of the third number “2” indicates the second operation of the second job, denoted as *O*_22_. Subsequent numbers have similar meanings. Thus, the sequence of operations “2-1-2-3-1” can be expressed by operations sequence: *O*_21_–*O*_11_–*O*_22_–*O*_31_–*O*_12_.

### 4.3. Machine Selection Part

Similar to the OS, the length of the chromosomes in the MS part is also the total number of operations. In the MS part in Figure 1, for example, for operation *O*_22_, since the number in its corresponding position is 3, it represents selecting the third machine to handle this operation. The number of machines depends on the problem itself, consequently, the value in the MS part is designed for representing the serial number of the machine processing the current operation.

The advantage of this OS-MS chromosome representation is that this method is more versatile, and it can simplify the logical structure of array processing, which is convenient for subsequent genetic operations.

### 4.4. Population Initialization

Population initialization is an important task of GA. Generally, an efficient initialization method can speed up the population convergence speed [30]. Currently, the mainstream scheme to initialize the MS is the global and local selection (GL) strategy came up by Zhang et al. [19], which is also used in this article of MS part. On the premise of pre-calculating the total processing time, the load of each machine is fully considered, and then the most suitable machine is selected to process for each operation. However, for the initialization of the OS, a random method is more commonly used in the published literature. Therefore, in this section, we propose two efficient strategies to initialize the OS for arranging the proper sequences for all jobs, which are described as follows:

1. Choosing the job with the most remaining operations (CRO)
2. 6-Dimensional variables to determine the search position (6D-VSP)

For CRO, a job with the most remaining operations is selected each time. The specific steps of CRO are as follows:

- Step 1: create an array to record each job selection, initialize each element to 0.
- Step 2: create an array of length *n*, initialize each element as the operations of each job.
- Step 3: select a job with the most remaining operations (MRO) in the array created in Step 2. If the same value exists, select a job randomly.
- Step 4: store the selected job number in the array created in Step 1. Decrement the value at the corresponding position of the array created in Step 2 by 1.
- Step 5: repeat steps 3 and 4 until all elements created in step 2 are 0.

**Figure 2:**
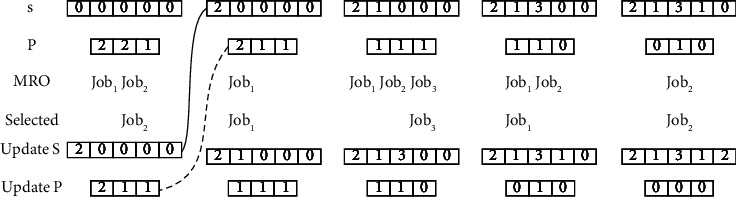
The process of CRO.

The implementation of CRO is shown in Figure 2. Take the 3 jobs in Table 1 with 5 operations as an example, the number of operations of each job is 2, 2, and 1, respectively. When the remaining operations are the same, a job is randomly selected. Assuming that the first job selected is Job_2_, then update the array *S*, while the value of the corresponding position of the array *P* is decreased by 1. Repeat this step until all values in array *P* are 0. Finally, the array *S* is the code for the OS part of the chromosome.

For the 6D-VSP, its essence is to increase the randomness of the selection of each job, avoiding the similarity of chromosomes in the initial stage. The specific steps of 6D-VSP are as follows:

- Step 1: determine two values that sum to 1 randomly to determine the lower bound (lb) and upper bound (ub).
- Step 2: generates an array of length the total number of operations, where the elements consist of job numbers.
- Step 3: the difference between the two values created in step 1 forms a new array of length total operations.
- Step 4: randomly generate an array between 0 and 1 with length equal to the total number of operations.
- Step 5: multiply the corresponding position elements (MCE) of the two matrices obtained in Steps 3 and 4 to obtain a new array.
- Step 6: add the elements in the array gotten in Step 5 to the lower bound determined in Step 1, and get an index array after sorting.
- Step 7: match the index array with the array generated in Step 2 to get a matched array.

**Figure 3:**
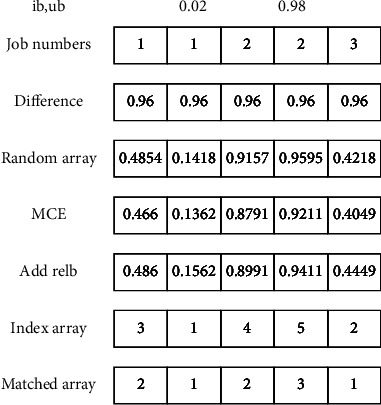
The process of 6D-VSP.

The implementation of 6D-VSP is shown in Figure 3. The array obtained by (8) is sorted to obtain an index array. From the position of each element of index array in the job numbers array, the matched array can be obtained, which is the coding of the OS part.

(8) $$\begin{array}{c} Random\;array*\left(ub-lb\right)+lb\text{.} \end{array}$$

The purpose of the two strategies is to reduce the search space during the initial optimizing process. The effectiveness of these strategies will be verified in the subsequent experiments in Section 5.1.

### 4.5. Selection Operation

The population reproduction of GA originated from the laws of nature is based on individual selection operations [31]. Excellent individuals are chosen from the initial population and retained in the mating pool to provide parental genes for the next generation of evolution through one or more reproductions. The roulette wheel selection and stochastic universal selection are two popular selection methods.

The former is a fitness-based selection method, whereas the latter is a completely random selection method [32]. The convergence rate of roulette selection is faster, but the population tends to fall into local optimum. The advantage of stochastic universal selection is that the diversity of the population is well maintained in the process of evolution, but the speed of convergence is slower.

Therefore, to trade off the exploration and the exploitation, this article adopts the tournament selection. Tournament selection is based on a return sampling strategy, where a certain number of individuals are taken from the population at a time and the best one is selected to enter the offspring population. This process is repeated until the size of the new population reaches the size of the original population. It overcomes the problem of directly destroying the selection method based on population diversity as fitness. This strategy makes the outstanding individuals have more opportunities to be preserved. Concurrently, it takes the relative fitness value as the criterion for selecting, rather than directly using the proportion of fitness. It not only avoids the influence of “super individual,” but also avoids the premature convergence [33].

### 4.6. Crossover Operation

Being a basic operation of GA, crossover operation plays a vital role in the optimization process of GA. The basic concept of the operation is that different chromosomes exchange some genes with each other, and then generate new chromosomes. Based on the double-layer chromosome structure introduced in Section 4.1, we design two different crossover operations, respectively, for OS and MS part.

**Figure 4:**
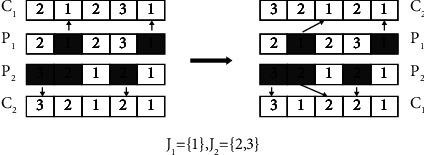
The process of POX.

In order to avoid generating invalid chromosomes and satisfy scheduling constraints of OS, we use precedence operation crossover (POX) [34] method. Take the five operations in Table 1 as an example to illustrate the process of generating two child individuals with POX. The implementation of POX is shown in Figure 4, and the detailed steps are as follows:

- Step 1: divide job set randomly into two sub-job sets *J*_1_ and *J*_2_ and select two individuals randomly as parent chromosomes *P*_1_ and *P*_2_
- Step 2: copy genes that belong to *J*_1_ from *P*_1_ and *J*_2_ from *P*_2_ to child individuals *C*_1_ and *C*_2_, keeping their position unchanged in *C*_1_ and *C*_2_
- Step 3: copy genes that belong to *J*_1_ from *P*_1_ and *J*_2_ from *P*_2_, then sequentially store in *C*_2_ and *C*_1_

**Figure 5:**
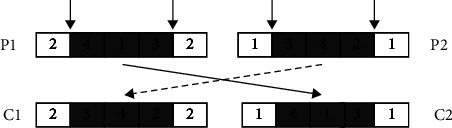
Machine selection crossover operation.

The MS part is a sequence of processing machines corresponding to each operation. The crossover operation in the MS section is machine re-selection, by swapping genes at two positions on the chromosome to generate two new sequences of MS. The number appearing in the same position on each chromosome indicates that this machine exists in the set of alternative machines. The new chromosomes generated by allele crossover will not change the previous constraints conditions. Therefore, the individual produced after the crossover operation is also a feasible scheme. In this article, the two-point crossover [35] method is adopted. The crossover process in the MS part is shown in Figure 5.

### 4.7. Mutation Operation

After the crossover operation, the newly generated chromosomes need to conduct a mutation operation, which is a popular phenomenon in the evolutionary process of species. When performing the mutation operation, one or more genes in a chromosome will be changed with a probability. The main objective of the mutation operation is to enhance the population diversity and to avoid the premature convergence of algorithm [36].

When performing the mutation operation in the OS part of a chromosome, two mutate positions need to be selected according to probability (pm). After that, the genes of the chromosome between the two positions are swapped. Hence, a new chromosome can be obtained. Note that, the new chromosome does not change the genes, but just repositions the genes, so the new scheme is also feasible.

The probability of mutation in the MS part is the same as in the OS part. If there is a mutation in the MS part of the chromosome, the new processing machine is randomly selected from the candidate machine set. The mutation steps of the MS part are as follows:

- Step 1: select individuals from the population in order
- Step 2: generate a probability value randomly, if the value is less or equal to the Pm, go to Step 3; otherwise, go to Step 1
- Step 3: determine a mutation point of MS part of the chromosome randomly
- Step 4: pick another machine from the set of candidate machines randomly at the mutation position to replace the original processing machine

### 4.8. Reinitialization of Part Population

Premature convergence is a popular phenomenon in GA. How to deal with the phenomenon is crucial for the exploration ability of GA. In this study, when the population falls into a local optimum, a small part of the population is reinitialized, and then, the diversity of the population can be improved. During this process, the new chromosomes generated by the reinitialization strategy randomly replace the same number of original individuals except for the best one that has been obtained. The newly formed population participates in subsequent evolution.

The number of times of reinitialization is completely adaptively adjusted according to the situation of current evolution. The more times the population gets trapped in a local optimum, the more times the strategy is executed. The evolution will not stop until the constraint conditions are reached, or the output has obtained the optimal solution.

After the reinitialization, many chromosomes trapped in local optima are replaced by some new randomly generated chromosomes, and then the population diversity can be improved. Thus, the exploration ability of the population can be further enhanced.

The GLRe terminates the evolution when the maximum number of generations is reached. The fitness of the best individual and the corresponding scheduling scheme is output.

### 4.9. Framework of the Improved GA

**Figure 6:**
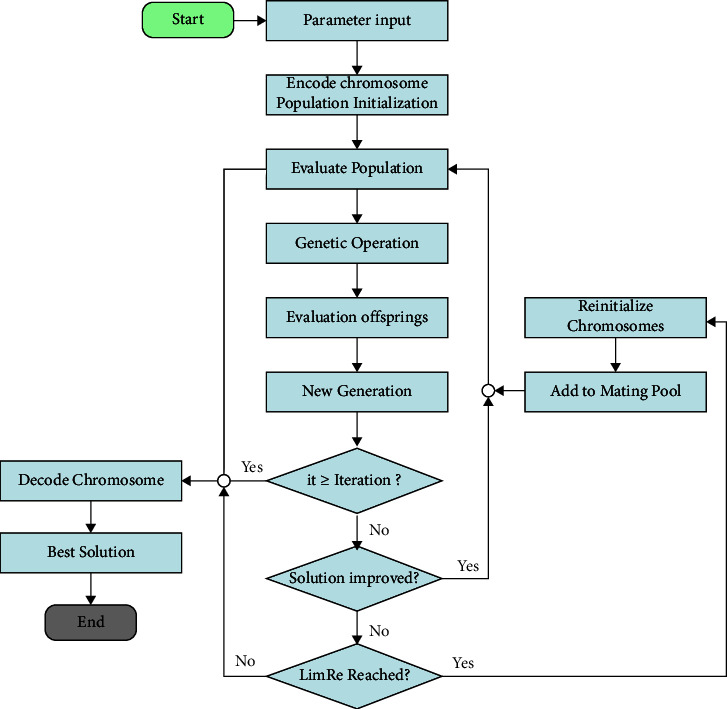
Flow chart of GLRe.

Based on the above discussions, the framework of the proposed algorithm can be demonstrated in Figure 6 and explained in Algorithm 1.

## 5. Experimental Results and Analyses

In this section, a series of comparison experiments are conducted to analyze the performance of GLRe in solving FJSP. In order to demonstrate the experimental results better, we use two sets of benchmark data for testing. The first is five small-scale Kacem [18] F-FJSP instances. The second data set is more complex instances from Brandimarte [37].

The proposed GLRe in this article is implemented in Matlab 2020a on Intel Core i5 with 8 GB main memory under Windows 10 system. Each instance will be run 20 times, other parameters of GLRe are as follows:

Population size (Pop): 5 × m × n.
Maximum number of generations (Num): 10 × m × n.
Rate of global selection: 0.6.
Rate of local selection: 0.3.
Rate of random selection: 0.1.
Rate of 6D-VSP: 0.9.
Tournament approach: *k* = 2.
Number of reinitializations:
  (9) $$\begin{array}{c} T=\left\{\begin{array}{cc} \frac{\sum_{it=x}^{x+100}bestfit}{100}=1\text{,} & x\in Num\text{,}\\ K+1\text{,} & K\in N\text{.} \end{array}\right. \end{array}$$
Generations limit before reinitialization:
  (10) $$\begin{array}{c} \lim\;=\left\{\begin{array}{cc} it>\frac{Num}{3}\text{,} & it\in Num\text{,}\\ \frac{\sum_{it=x}^{x+100}bestfit}{100}=1\text{,} & x\in Num\text{.} \end{array}\right. \end{array}$$

### 5.1. Effectiveness of Improvement Strategies

In this part, we use 10 instances of Brandimarte date for testing. The algorithm combining the three strategies of CRO, 6D-VSP, and Reinitialization is called Re. GA-GS (Global selection combined with GA), GA-GSRe (Global selection and Re combined with GA), GA-GL (Global and Local selection combined with GA), and conventional GA and proposed algorithm GLRe (GL combined with Re) are compared to prove the effectiveness of the improved strategy.

We compare the optimal solutions and average values of these five algorithms and their respective deviations. The degree of dispersion is expressed by the relative percentage deviation (RPD) [38]. Tables 2 and 3, show all the experimental results. The first column of the two tables contains 10 different data instances. Best and average represent the optimal and average values of makespan obtained by algorithms, respectively. LB denotes the lower bounds of Brandimarte instances, which are obtained from the currently known literature [11]. The best RPD (RPDB) values of all instances are calculated by the five algorithms according to formula (11) shown in columns 3–7 of Table 2. Columns 8–12 in Table 3 are the average RPD (RPDA) values calculated by formula (12) for each instance in Table 2. The best result for each instance is shown in bold.

(11) $$\begin{array}{c} RPDB=\frac{C_{m}-LB}{LB}\times 100\text{,} \end{array}$$

(12) $$\begin{array}{c} RPDA=\frac{AV-LB}{LB}\times 100\text{.} \end{array}$$

For the best and RPDB from Tables 2 and 3, GLRe is superior to GA-GS, GA-GSRe, GA-GL, and conventional GA. For average and RPDA, GLRe achieves an overwhelming advantage over its competing algorithms from the smaller scale MK01 to the larger scale MK10. By controlling the fusion of different algorithms, the experimental result proves that the GLRe consistently gives high-quality solutions for FJSP.

In order to exclude the interference of other contingent factors and further verify the role of each strategy in the algorithm, we select MK03 and MK08 instances where each algorithm can converge for testing.

**Figure 7:**
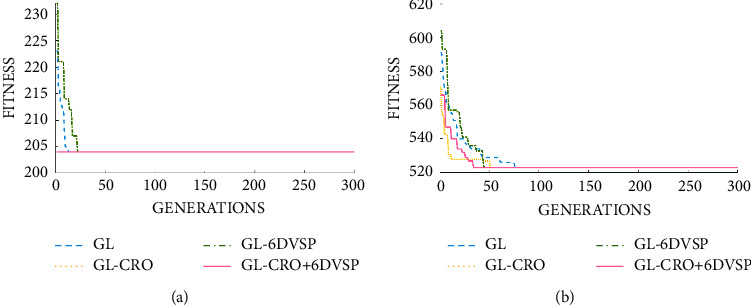
Comparison between four algorithms on MK03 and MK08 instances. (a) MK03. (b) MK08.

Through the previous experimental verification, we have come to the conclusion that the global selection and local selection algorithm (GL) are effective. On the basis of the GL, we add the strategies of CRO (GL-CRO), 6D-VSP (GL-6DVSP), and the combination of CRO and 6D-VSP (GL-CRO+6D-VSP). The experimental results are shown in Figure 7.

As can be seen from Figure 7, the initial solution obtains a smaller initial value than the original GL after adding the strategy CRO. Due to the small scale of the MK03 problem instance, the graph appears as a straight line, that is, the optimal scheduling scheme is obtained at the beginning. At the same time, this also means that the strategy effectively improves the search space of the algorithm.

After adding the 6D-VSP on the basis of the original GL, it can be seen that the initial value has a certain increase compared with GL. The reason for this phenomenon can be explained that the addition of the 6D-VSP increases the disorder of sorting, which can well reduce the probability of the population falling into the local optimum during the evolution process.

At the same time, we can observe that after combining the CRO and 6D-VSP strategies, not only the initial solution space is reduced, but also the optimal scheduling sequence is found in the first generation.

After testing an instance with a larger problem scale, MK08, we found that the above features are preserved. Benefiting from the feature that 6D-VSP can increase the sequence disorder, the population can evolve more quickly to obtain the optimal scheduling scheme while avoiding falling into local optimum.

For the Reinitialization strategy, both MK03 and MK08 can converge, and there is no specific reference. The Reinitialization strategy only works in the later stages of algorithm evolution. Therefore, we combine the three strategies and select MK09 and MK10 with a larger problem scale for testing. Combining the three strategies to test the performance of Re does not affect the existing conclusions.

**Figure 8:**
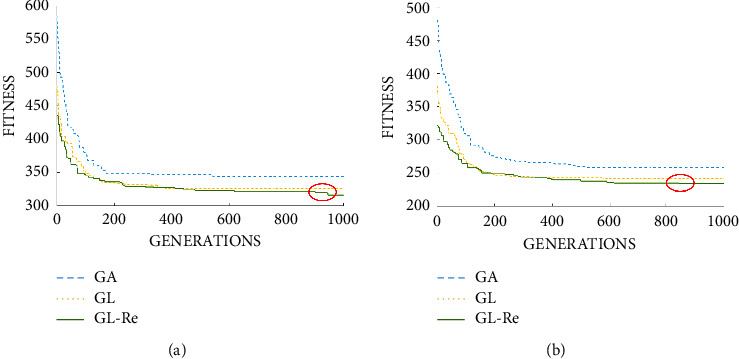
Comparison between GA, GL, and GLRe on MK09 and MK10 data instances. (a) MK09. (b) MK10.

From Figure 8, the above-obtained search space is smaller and the characteristics of faster convergence are still verified. Meanwhile, we can see that after many generations, the diversity of the population decreases seriously, the chromosome genes tend to be consistent, and the other two algorithms basically stop evolving. In our GLRe, this situation has been significantly improved by introducing the concept of reinitialization. In the later stages of the evolution, the algorithm can also approach the optimal solution gradually. Under the same number of generations, the convergence speed of the proposed GLRe is faster, followed by GL. The convergence speed of GA is relatively slow. Meanwhile, we can observe that the final solution of our algorithm is better.

To further verify the effectiveness of the above strategies, we conduct independent tests on these three strategies, respectively. Each instance is tested 20 times and compared with the average data of GA-GL in Table 2. Count the number of occurrences of the above features for each algorithm separately, and calculate their frequency. After the total number of tests (Tnum) for each strategy reaches two hundred, the experimental results are recorded. The experimental results are shown in Table 4, and the statistical results are shown in Table 5.

As can be seen from Tables 4 and 5, CRO reduces the search space of the algorithm by 100 percent in the initialization phase. The performance of the 6D-VSP strategy is unstable, and the efficiency of the strategy will be affected by different problem instances. The number of experimental features still appears more than half, and it can be inferred that the 6D-VSP strategy is still effective. When the problem size is small, the effectiveness of the Re strategy cannot be sufficiently demonstrated. However, as the size of the problem increases, the newly added chromosomes expand the gene pool, preventing the population from falling into a local optimum to a certain extent.

Combining the results of the above experiments, we can affirm that the three strategies play an active role in the algorithm.

### 5.2. Comparison with Other Algorithms

In this part, to testify the comprehensive performance of GLRe, five state-of-art algorithms are selected as peer algorithms, that is, SLGA [23], edPSO [10], GWO [11], HA [38], and MACROG [39]. The experimental parameter settings are the same as those described in the previous section.

In SLGA, the author innovatively combines reinforcement learning with a genetic algorithm, and uses Q-learning as a self-learning model. edPSO is a neighborhood-based genetic algorithm that proposes a hybrid clustering model. GWO is a new swarm intelligence algorithm inspired by social hierarchy and the hunting behavior of gray wolves. HA generates and evaluates different solutions by changing the weights of his strategies. A new decentralization model is proposed in MACROG. MACROG−FJSP is an improved version of the greedy algorithm.

The above algorithms combined machine learning, unsupervised learning, new swarm intelligence algorithm, variable weighting method, decentralized model, and greedy strategy to propose effective algorithms, respectively. Each algorithm is representative of the field, which is the reason why these algorithms are chosen for comparison in this article.

Experimental results of the two sets of benchmark data, in terms of makespan, are shown in Tables 6 and 7, respectively. The best results for each instance are shown in bold. The symbol “—” in the table indicates that the instance is not solved in the literature.

From the comparison results on the Kacem data set demonstrated in Table 6, we can see that both GLRe and GWO can get the best results on 4 out of the 5 instances. Moreover, GLRe obtains LB results on 3 instances, while GWO yields 4 LB results. The comparison results indicate that our algorithm performs well on small-scale problems.

In order to verify the algorithm's ability to address medium to large-scale problems, we used 10 more complex Brandimarte instances to test the performance of GLRe. Experimental results measured by makespan are shown in Table 7. From the table, we can observe that GLRe, edPSO, and SLGA display the best performance on 5 out of the 10 large-scale instances. On the contrary, GWO only attains the best result on two instances though it offers the most outstanding performance on the Kacem instances. Furthermore, HA also cannot exhibit favorable performance on the Bandimarte instances though it offers very promising performance on the Kacem instances. The results manifest that GLRe is more suitable for large-scale problems than small-scale problems.

Table 8 gives the RPD values (%) of Table 7. In terms of RPD, it can be observed from the table that GLRe can figure out the best solutions on MK01, MK03, MK07, MK08, and MK10. Also, it can provide the second best results measured by RPD on 3 out of the 5 remaining instances. Furthermore, regarding the mean value of RPD, GLRe is the lowest among all algorithms, which indicates that our algorithm performs better on medium and large FJSPs.

**Figure 9:**
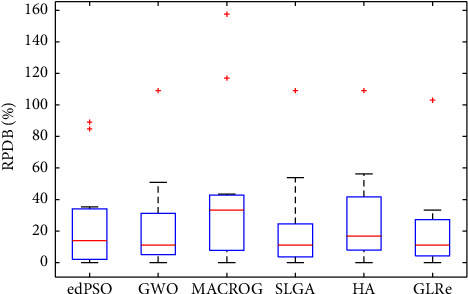
Boxplot of RPDB for each algorithm in Table 8.

The RPD boxplot of the six algorithms in Table 8 is shown in Figure 9, which can further verify the performance of our algorithm. From the results in the figure, we can clearly observe that the median and range of RPD values of GLRe are smaller. Meanwhile, besides SLGA, the results obtained by GLRe are relatively concentrated, which indicates that GLRe has good performance in solving FJSP. The results are consistent with our previous conclusions.

Table 9 shows the actual number of instances solved (AS) for all compared algorithms and the amended-RPD (aRPD) for each algorithm. The value of aRPD is calculated as follows:

(13) $$\begin{array}{c} aRPD=\frac{\left\{\overset{AS}{\underset{i=1}{\sum }}\left(Best-LB\right)\times 100/LB\right\}}{AS}\text{,} \end{array}$$

where Best represents the best result after 20 runs for each instance of these algorithms. The column defined as “improvement” in the table represents the reduced value of aRPD obtained by GLRe relative to other algorithms of two groups of benchmarks. Except for the SLGA, GLRe has different degrees of improvement compared to others. However, the aRPD of our algorithm is slightly better than SLGA. According to the comparison results of the above experiments, we can come to a conclusion that GLRe has better performance and efficiency in minimizing the makespan of FJSP.

Friedman test is a statistical test method proposed by Friedman in 1973 for the homogeneity of multiple (correlated) samples [40]. To better compare the performance of our GLRe with other algorithms, we divide the test into three parts. Take the smaller MK01-05, Kacem01, and Kacem02 as a group, the larger problem scale MK06-10 as a group, and finally the entire 12 datasets as a large group. The results of the Friedman test are shown in Table 10. From the table, we can observe that GLRe has a gap with other algorithms in solving small-scale problem instances MK01-05, Kacem01, and Kacem02. However, as the complexity of the problem instances increases, GLRe shows superior performance in solving MK06-10 instances. Overall, our proposed GLRe has the best average ranking and highest priority compared to the RPDB values of the other five algorithms. This test result is consistent with the previous conclusion and further validates GLRe's excellent performance relative to its competitors in solving FJSP.

## 6. Conclusion and Future Work

In this article, we proposed an improved hybrid algorithm combing global and local search with reinitialization (GLRe) with GA-based for solving the flexible job shop scheduling problem (FJSP). Considering distinct properties of FJSP, a double-layer gene chain chromosome representation method was proposed. Based on the chromosome representation, genetic operations can be independently carried out on the operation sequence and the machine selection. Moreover, to improve search efficiency, a new initialization approach was proposed in which a method combining choosing the job with the most remaining operations (CRO) and 6-dimensional-variable determination of the search position (6D-VSP) was added to determine the initialization of operation sequence. This operation sequence aimed at improving the overall quality of the initial solution and reducing the global search space. When the population falls into local optimum, some chromosomes are reinitialized. Thus, chromosome diversity can be improved and the gene pool of the population is enriched.

The algorithm had been extensively tested on the selected benchmarks test set, and compared with other algorithms in the literature. The experimental results showed that GLRe has better convergence speed and higher solution quality, especially on large-scale FJSP.

Although GLRe performs well in solving FJSP, the settings of some genetic parameters are not deeply considered and the performance of GLRe for solving other scheduling problems is unknown. In future work, we will continue to deeply study the influence of different parameters on GLRe for solving FJSP, and focus on the application of GLRe to other combinatorial optimization problems.

## Figures and Tables

**Algorithm 1:**
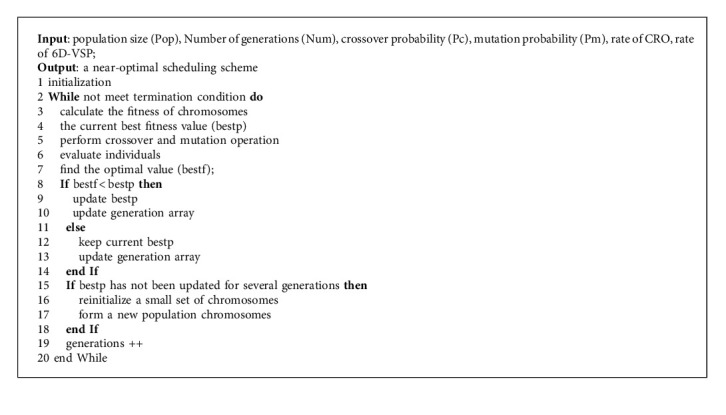
GLRe.

**Table 1:** A simple instance of 3 × 4 P-FJSP.

Job	Operation	*M*_1_	*M*_2_	*M*_3_	*M*_4_
Job_1_	*O*_11_	—	3	2	5
	*O*_12_	2	—	3	3

Job_2_	*O*_21_	3	—	4	2
	*O*_22_	2	1	3	5

Job_3_	*O*_31_	-	3	—	5

**Table 2:** Makespan of best and average for each algorithm.

Dataset instance	LB	Best					Average				
		GA-GS	GA-GSRe	GA-GL	GA	GLRe	GA-GS	GA-GSRe	GA-GL	GA	GLRe
MK01	36	40	40	40	44	**40**	41.4	41.4	41.9	46.5	**40.9**
MK02	24	29	29	29	36	**29**	29	29.1	29.1	37.1	**29.3**
MK03	204	204	204	204	204	**204**	204	204	204	228.9	**204**
MK04	48	63	63	64	83	**62**	66.9	65.9	66	85.4	**65**
MK05	168	177	177	176	191	**176**	182.8	180.6	179.9	193.1	**178.5**
MK06	33	66	67	65	81	**65**	69.4	68.8	68.4	84.7	**68**
MK07	133	151	151	149	178	**146**	155.6	156.3	151.7	181.6	**150.2**
MK08	523	523	523	523	523	**523**	523	523	523	551.2	**523**
MK09	299	319	313	311	348	**311**	324.8	325.8	322.1	359.3	**315.4**
MK10	165	224	230	227	310	**220**	236	237.9	236	323.5	**227.4**

**Table 3:** Comparison of RPDB and RPDA for each algorithm.

Dataset instance	RPDB					RPDA				
	GA-GS	GA-GSRe	GA-GL	GA	GLRe	GA-GS	GA-GSRe	GA-GL	GA	GLRe
MK01	11.1	11.1	11.1	22.2	**11.1**	15	15	16.4	29.2	**13.6**
MK02	20.8	20.8	20.8	50	**20.8**	20.8	21.3	21.3	54.6	**22.1**
MK03	0	0	0	0	**0**	0	0	0	12.2	**0**
MK04	31.3	31.3	33.3	72.9	**29.2**	39.4	37.3	37.5	77.9	**35.4**
MK05	5.4	5.4	4.8	13.7	**4.8**	8.8	7.5	7.1	14.9	**6.3**
MK06	100	103	96.9	145.5	**96.9**	110.3	108.5	107.2	156.7	**106.1**
MK07	13.5	13.5	12	33.8	**9.8**	16.9	17.5	14.1	36.5	**12.9**
MK08	0	0	0	0	**0**	0	0	0	5.4	**0**
MK09	6.7	4.7	4	16.4	**4**	8.6	8.9	7.7	20.2	**5.5**
MK10	35.8	39.4	37.6	87.9	**33.3**	43	44.2	43	96.1	**37.8**

**Table 4:** Statistics of occurrences of each feature.

Strategy	Instance									
	MK01	MK02	MK03	MK04	MK05	MK06	MK07	MK08	MK09	MK10
CRO	20	20	20	20	20	20	20	20	20	20
6D-VSP	5	10	20	14	17	11	9	20	12	14
Re	5	7	0	8	4	9	12	0	16	18

**Table 5:** The probability of each feature of the three strategies.

Strategy	Tnum	Count	Frequency (%)
CRO	200	200	100
6D-VSP	200	134	67
Re	200	79	39.5

**Table 6:** The makespan of Kacem instance.

Dataset instance	LB	edPSO	GWO	MACROG	SLGA	HA	GLRe
Kcaem01	11	**11**	**11**	**11**	**11**	**11**	**11**
Kcaem02	14	17	**14**	20	**14**	15	**14**
Kcaem03	11	—	**11**	14	**11**	13	**11**
Kcaem04	7	8	**7**	—	—	**7**	8
Kcaem05	11	—	13	19	—	**12**	**12**

**Table 7:** The makespan of Bandimarte instance.

Dataset instance	LB	edPSO	GWO	MACROG	SLGA	HA	GLRe
MK01	36	41	**40**	**40**	**40**	42	**40**
MK02	24	**26**	29	32	27	28	29
MK03	204	207	**204**	**204**	**204**	**204**	**204**
MK04	48	65	64	64	**60**	75	62
MK05	168	**171**	175	179	172	179	176
MK06	33	**61**	69	85	69	69	67
MK07	133	173	147	172	**144**	149	**144**
MK08	523	**523**	**523**	552	**523**	555	**523**
MK09	299	**307**	322	421	320	342	311
MK10	165	312	249	358	254	242	**220**

**Table 8:** The RPDB values (%) of Table 7.

Dataset instance	edPSO	GWO	MACROG	SLGA	HA	GLRe
MK01	13.9	**11.1**	**11.1**	**11.1**	16.7	**11.1**
MK02	**8.3**	20.8	33.3	12.5	16.7	20.8
MK03	1.5	**0**	**0**	**0**	**0**	**0**
MK04	35.4	33.3	33.3	**25**	56.3	29.2
MK05	**1.8**	4.2	6.5	2.4	6.5	4.8
MK06	**84.8**	109.1	157.6	109.1	109.1	103
MK07	30	10.5	29.3	**8.3**	12	**8.3**
MK08	**0**	**0**	5.5	**0**	6.1	**0**
MK09	**2.7**	7.7	40.8	7	14.4	4
MK10	89.1	50.9	117	53.9	46.7	**33.3**
Mean	26.8	24.8	43.4	22.9	26.8	**21.5**

**Table 9:** The comparison of aRPD.

Algorithm	AS	aRPD (%)	GLRe's aRPD (%)	Improvement (%)
edPSO	13	23.3	17.6	5.7
GWO	15	17.7	15.9	1.9
MACROG	14	41.2	16.0	25.3
SLGA	13	17.6	16.5	1.1
HA	15	21.3	15.9	5.4
GLRe	15	15.9		

**Table 10:** Friedman test results for 6 algorithms.

Algorithm	MK01-05, Kacem01, Kacem02	Priority	MK06-10	Priority	Value of the mean rank		Final priority
edPSO	3.79	4	3.10	3	3.50		4
GWO	3.14	3	3.30	4	3.21		3
MACROG	4.29	5	5.60	5	4.83		6
SLGA	2.29	1	3.00	2	2.58		2
HA	4.43	6	4.20	6	4.33		5
GLRe	3.07	2	1.80	1	2.54		1

## Data Availability

The experimental data used to support the findings of this study are available from the corresponding author upon request.
